# Subsequent primary neoplasms after childhood cancer therapy – design and description of the German nested case–control study STATT-SCAR

**DOI:** 10.1007/s10552-023-01760-5

**Published:** 2023-08-02

**Authors:** Peter Scholz-Kreisel, Cornelia Becker, Melanie Kaiser, Seyed Hamidreza Mahmoudpour, Mathias Voigt, Meike Ressing, Maria Blettner, Gabriele Calaminus, Katja Baust, Cathy Scholtes, Martin Zimmermann, Sylke Ruth Zeissig, Heinz Schmidberger, Heiko Karle, Sarah Meyer-Oldenburg, Peter Kaatsch, Claudia Spix

**Affiliations:** 1 Institute of Medical Biostatistics, Epidemiology and Informatics, University Medical Center of the Johannes Gutenberg-University Mainz, Mainz, Germany; 2 Federal Offices for Radiation Protection, Neuherberg, Germany; 3 German Childhood Cancer Registry (GCCR) Division of Childhood Cancer Epidemiology at the Institute of Medical Biostatistics, Epidemiology and Informatics (IMBEI), University Medical Center of the Johannes Gutenberg-University Mainz, Mainz, Germany; 4 Department of Pediatric Hematology and Oncology, University Hospital Bonn, Bonn, Germany; 5 Department for Pediatric Hematology and Oncology, Children’s Hospital, Medical School Hannover, Hannover, Germany; 6 Institute of Clinical Epidemiology and Biometry (ICE-B), University of Würzburg, Würzburg, Germany; 7 Regional Centre Würzburg, Bavarian Cancer Registry, Bavarian Health and Food Safety Authority, Würzburg, Germany; 8 Department for Radiation Oncology and Radiotherapy, University Medical Center of the Johannes Gutenberg-University Mainz, Mainz, Germany

**Keywords:** Childhood cancer, Subsequent primary neoplasm, Radiotherapy, Chemotherapy, Nested case control study

## Abstract

**Background:**

Subsequent primary neoplasms (SPN) are among the most severe late effects and the second most frequent cause of death in childhood cancer patients. In this paper we introduce method and properties of the STATT-SCAR study (Second Tumor After Tumor Therapy, Second Cancer After Radiotherapy), which is a joint nested matched case–control study to evaluate the impact of chemotherapy (STATT) as well as radiotherapy (SCAR) on the risk of developing a SPN.

**Methods:**

Based on the cohort of the German childhood cancer registry (GCCR), we selected patients diagnosed with a first neoplasm before age 15 or younger between 1980 and 2014. We selected those with a SPN at least half a year after the first neoplasm, and matched up to four controls to each case. Therapy data were acquired from various sources, including clinical study centers and treating hospitals. To analyze the impact of radiotherapy, organ doses were estimated by using reconstructed treatment plans. The effect of chemotherapy was analyzed using substance groups summarized after isotoxic dose conversion.

**Results:**

1244 cases with a SPN were identified and matched with 4976 controls. Treatment data were acquired for 83% of all match groups (one case and at least one control). Based on preliminary analyses, 98% of all patients received chemotherapy and 54% of all patients were treated with radiotherapy.

**Conclusions:**

Based on our data, detailed analyses of dose response relationships and treatment element combinations are possible, leading to a deeper insight into SPN risks after cancer treatments.

**Trial registration:**

The study is registered at the German clinical trial register (DRKS) under number DRKS00017847 [45].

## Introduction

Currently 82% of all childhood cancer patients in high income countries survive for at least 15 years [1], contributing to a growing population of long-term survivors. Even after former cancer patients are considered, or consider themselves “cured”, they are likely to be affected by a large number of potentially severe late effects [2, 3]. Subsequent primary neoplasms (SPN) are among the most common late effects and the second most common cause of death in childhood cancer survivors [4, 5]. Studies found an up to 19-fold increased risk for death due to a SPN when comparing childhood cancer survivors to the general population [4]. Similar studies also discovered a clear association between treatment of the first neoplasm and the risk of developing a SPN [6–8]. However, to date there are relatively few publications presenting continuous dose response relationships and considering combinations of treatment components in a multiple model. Some of them included all first and second primaries combined [6, 7, 9], while others restricted the study population to leukemia as subsequent primaries [8, 10] or to solid tumors as first [10] or subsequent [11] neoplasms. Some of these studies found an increased risk for a SPN when alkylating agents were administered to treat the primary tumor [6–8], while others did not find an increased risk [9] or only for sarcoma as SPN [11]. As to platinum compounds [6–9], epipodophyllotoxins [6, 7, 9, 10], anthracyclines [6, 7, 9, 11] and vinca alkaloids [6, 8, 9], results were ambiguous with only one or two studies, respectively, presenting a significantly increased risk for SPN for these substance groups in a multiple model. For antimetabolites [6, 8, 9] and asparaginase [6, 9], studies did not find any increased risk or even a decreased risk [9] for subsequent primaries. Regarding radiotherapy, most studies found a clear association between radiation exposure and especially solid SPN [12–14].

Findings for combined effects of chemotherapeutics and radiotherapy were ambiguous. While some studies showed a higher incidence of SPNs for combined chemo-radiotherapy compared to either treatment alone [15], others did not [12], or even found a reduction of SPN incidence for certain combinations [16]. Regarding the dose–response relationships most studies assumed a linear or at least a monotonous relationship between radiation dose and the SPN risk [14]. Some more recent publications, however, found a declining relative excess risk for some SPN sites when treated with very high doses [17, 18].

The goal of the STATT-SCAR-study (Second Tumor After Tumor Therapy-Second Cancer After Radiotherapy) is to estimate dose response curves for all treatment components used for the first primary and the risk of developing a SPN. In this paper, we describe the study design, data acquisition and selection procedures, selected methods for planned future analyses, the matching process, and report first descriptive results of the STATT-SCAR study.

## Methods

### Study design

This is a nested matched case–control study within the German Childhood Cancer Registry with retrospective exposure acquisition.

The German Childhood Cancer Registry routinely and systematically registers childhood cancer patients with a diagnosis defined by the International Classification of Childhood Cancer (3rd edition) [19], who resided in Germany at the time of diagnosis since 1980. Registration generally used full identifying information and requires the consent of parents and/or patients. SPN occurring before the 18th birthday treated in pediatric oncology are reported to the GCCR by the treating hospitals. The close cooperation with the clinical study groups (see below) ensured later occurring SPNs were made known to the GCCR, particularly those occurring within the organized follow-up of the studies. Beyond this, the GCCR contacts all patients or families regularly about every 2–5 years asking about events and SPN. Patient reported SPN were validated with the help of patient provided records, the treating hospital, or the treating physician, provided the patient consents. The SPNs were registered independent of the patient’s age. Registration of especially late SPN was likely somewhat incomplete, which is, however, not a major issue for a case–control design. Information on cancer predisposition syndromes is available, but was not recorded systematically.

For the current study, we selected patients diagnosed with a first neoplasm before age 15 between the years 1980–2014. Patients had to be registered resident in Germany at the time of both the diagnosis of the first and second neoplasm. The minimum latency between the two diagnoses to be included in the study was set to 6 months, which was also the minimum required survival time.

Additionally, we excluded 18 patients, for whom the SPN was considered an auxiliary diagnosis or directly FPN related. We identified 1244 SPN cases as suitable for the analysis and matched them with at least one and up to four controls each out of a pool of 54,420 eligible patients [20]. We used risk-set-sampling [21], which allowed a case to be included as a control for another case depending on the timing of its SPN and to be sampled as a control more than once.

We matched the controls as closely as possible according to the age and year at FPN diagnosis (at most ± 5 years), sex, and recorded SPN-free survival of the corresponding case. An ad hoc match score defined as (difference in age (days) + difference in birthdate (days) + difference in diagnosis date (days)) was used to select the four patients with the lowest scores as controls.

### Treatment data acquisition

**Fig. 1:**
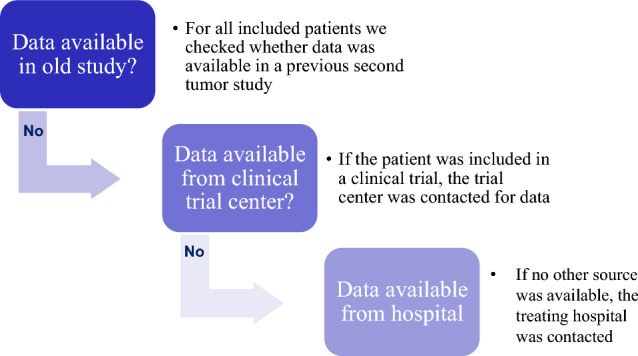
Data source selection process for patients included in STATT-SCAR. (GPOH: Gesellschaft für Pädiatrische Hämatologie und Onkologie (Society for Pediatric Hematology and Oncology))

Treatment data were acquired from different sources (Fig. 1). Firstly, we reused the data from a previous study conducted at the German Childhood Cancer Registry in 2009 [6], which used individual data from treating hospitals and supplemented it with treatment protocol information from clinical therapy studies if incomplete (mixed source data). Further treatment data were not available at the German Childhood Cancer Registry directly but were accessed as part of the long-standing cooperation with the nationwide clinical therapy study groups of the German Society for Pediatric Oncology and Hematology (GPOH), which ensured pediatric oncology patients are treated according to a nationwide protocol [22]. Clinical therapy study groups existed for most tumor sites. In the early 1980s about 60% of all German childhood cancer patients were treated according to such a protocol; by 2014 this percentage had increased to about 95% [22]. All major treatment protocols with their respective treatment arms, cumulative administered substance and irradiation doses per treatment arm were collected in this database. The allocated treatment arm is available at the respective study center for most protocol patients (intention to treat (ITT)-data). Some study groups provided individual treatment data or individual protocol deviations (“as treated”-data). The remaining data, especially for non-protocol patients, were acquired from the treating hospitals (“as treated”-data). In some cases, this was incomplete but could be supplemented with protocol information, if a protocol was mentioned in the records (mixed source data). As particularly the hospital data acquisition was costly and time consuming, it was applied only for cases and match groups with no control data yet.

For some patients, data were available from more than one source, which will provide some insight into reliability of especially ITT-data.

### Ethics and data protection

The baseline German Childhood Cancer Registry data and the data from the study groups required consent, but this was already included in the consent forms for the clinical treatment. Data from hospitals were obtained pseudonymized via a trust center (Cancer Registry of Rhineland-Palatinate). All acquired data was finally pseudonymized by the trust center to disable linking this data with the Childhood Cancer Registry Data.

**Fig. 2:**
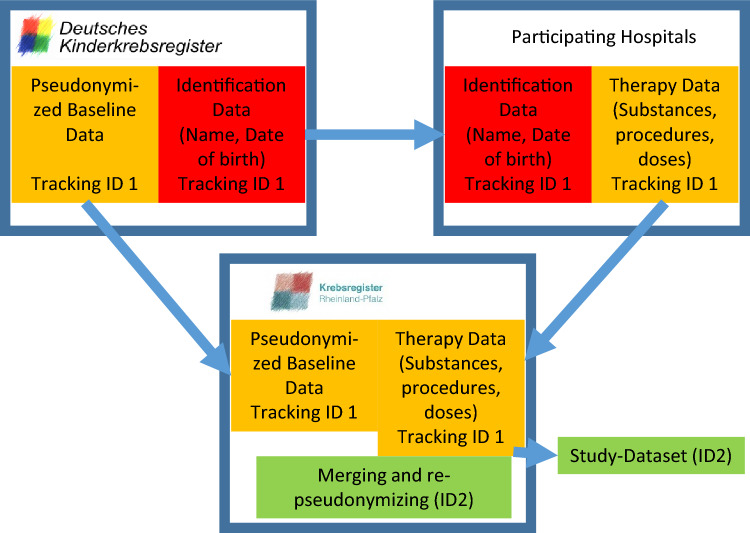
Data pseudonymization using a trust center. To prevent de-pseudonymization by the study or the data providers, an independent trust center was necessary to re-pseudonymize the data before passing them to the STATT-SCAR-study

The study was approved by the ethical committee of Rhineland-Palatinate (No. 837.280.15). The trust center procedure was developed in cooperation with the data protection officer of the University Medical Center Mainz (Fig. 2).

### Dosimetry for radiotherapy data

Radiation dosimetry was performed in cooperation with the Department for Radiation Oncology and Radiotherapy at the University Medical Center Mainz. For each tumor entity standard irradiation plans, based on tumor type and site, patients age, patients sex and year of treatment were redesigned retrospectively. A precise treatment plan according to the respective radiotherapy manual was created for patients for whom we had exact information about the location and size of the tumor. If no radiation manual was available, it was assumed that the patient was treated according to best clinical practice. If the exact location remained unknown, a proxy-location using the ICD-O-3 topology code and the most frequent location data were used for FPN, SPN or both. These irradiation plans were then used to simulate treatment in the Eclipse V.13.1 therapy planning software (Varian Medical System, Palo Alto, CA). For dose estimation the University of Florida hybrid phantoms family was used [23, 24]. Dose volume histograms (DVHs) were estimated using the Eclipse software version of the Analytical Anisotropic Algorithm for irradiation with a 6-MV linear accelerator or a CO-60 source. DVH data were exported and processed with the R package DVHmetrics Version 0.3.7, 2017. The calculated mean organ dose was used for further analyses.

### Doses for chemotherapy data

Cumulative doses were available per patient up to the date of diagnosis of the SPN of the respective index case minus an effect-latency period ranging from 2 months to 5 years, (see statistical analysis). This can mean that the same individual can enter the study with different doses of substances or radiation if it serves as control for multiple cases with different dates of diagnosis of the index case.

As there was a large observed number of different substances (almost 50), we grouped them based on the Anatomical Therapeutic Chemical (ATC) Classification [25]. We identified the following substance groups: alkylating agents, anthracyclines, antibiotics except anthracyclines, antimetabolites, corticosteroids, enzymes (asparaginase), epipodophyllotoxins, platinum derivates, topoisomerase inhibitors (other than epipodophyllotoxins), and vinca alkaloids. The approach in the previous study [6], inspired by Tucker et al. [26], was to use a category score per group. However, information is lost by categorizing. For the primary dose–response analysis, we determined conversion factors with respect to a reference agent per substance group and used these to calculate cumulative doses of reference-substance equivalents. Such conversion factors are available from the literature and have previously been used [10, 27–33] (see Table 1). When multiple factors were available from the literature, they were very similar, even when based on different kinds of isotoxicity, e.g. cardiotoxicity or haematotoxicity, or different kinds of equipotency in terms of the desired antitumor efficacy [34]. It can therefore be assumed that they can also be used for carcinogenicity in the context of subsequent neoplasms. Where no conversion factors were available in the literature, we supplemented these by conversion factors based on typical doses from a comprehensive list of treatment protocols of the GPOH from the years 1970 to 2018 [34]. A possible alternative approach was based on “equimolar doses”, as previously used by Van Dalen et al. and Vu et al. [35, 36]. This approach did not correlate with isotoxicity or typical doses [34].

**Table 1:** Examples for conversion of drug doses using external equivalence ratios

Substance group	Drug	Conversion factor	Reference drug	Reference
Alkylating agent	Cyclophosphamide	1	Cyclophosphamide	
Alkylating agent	Ifosfamide	0.244	Cyclophosphamide	(30)
Alkylating agent	Procarbazine	0.857	Cyclophosphamide	(30)
Anthracycline	Doxorubicin	1	Doxorubicin	
Anthracycline	Idarubicin	5	Doxorubicin	(32)
Platinum derivate	Cisplatin	1	Cisplatin	
Platinum derivate	Carboplatin	0.25	Cisplatin	(9)

### Statistical analysis

All analyses were tested for completeness and plausibility. For missing treatment data, an imputation process was developed. Imputation was performed for cases and for controls, where no other control with acquired data was available in the match group to keep the imputed data at a minimum. Complete case analysis was performed as a sensitivity analysis.

Conditional logistic regression was used to estimate Odds Ratios for a treatment effect on SPN risk. Dose response curves were determined using fractional polynomials with spike-at-zero [37–39]. As almost all patients received some form of combination treatment, we were planning to follow a specific selection process to choose from multiple models with the aim to find a good prediction model [40]. This enables statements about combinations of FPN treatment carrying a particularly high risk. Age at FPN diagnosis, age at SPN diagnosis, year of FPN diagnosis, latency, sex, type of first neoplasm and doses by treatment phase, especially long-term treatments, can be then investigated as potential effect modifiers.

To predict the potential power of the study, based on available preliminary ITT-data, we assumed a normal distribution for a hypothetical chemotherapeutic agent dose. Radiotherapy entered these scenarios as a confounder with varying assumptions about the strength of the radiotherapy effect and the correlation of radiotherapy with chemotherapy. From the available data we expected statistically significant (5%-level) odds ratios of 1.3 or more per 1 standard deviation of the exposure with at least 80% power. This is sufficiently powerful to allow for subset analyses as well, especially as effects reported in the literature are often larger.

In all chemotherapy models the binary (yes/no) variable radiation exposure was included as a potential confounder for chemotherapy.

In the next step, radiation dose was planned to be included as a continuous variable. When organ doses are unavailable, the “prescribed tumor dose by body area” will be used. Linear as well as linear quadratic models will be analyzed for best fit of data. Chemotherapy as a potential confounder for radiotherapy will also be analyzed as a binary variable (yes/no) using main substance groups (see above), as the use of individual drugs would have seriously affected model parsimony. Other planned subset analyses will include stratification by frequent FPNs and SPNs. Analyses by FPNs will require selecting controls and sometimes re-matching.

We were planning sensitivity checks, such as estimating results including/excluding imputed data, analyses by data source (ITT, as treated, mixed), or using the data source as an effect modifier. We are also going to explore different lengths of minimum latencies (i.e., excluding cases with SPN closer to the FPN than the minimum latency) and therapy-latencies (i.e., excluding exposures closer to the SPN than the minimum treatment-latency). However, longer latency periods will lead to smaller datasets and less power, e.g., a latency of 5 years leads to the exclusion of about 20% of the available data and more than half of the SPN with AML (acute myeloid leukemia). As further sensitivity analyses, we are planning to use different conversion methods for summarizing the agents from some of the substance groups (see above) to assess the differences.

As especially ITT-data are likely to measure the true exposure with an error, error estimation, and correction of treatment data are performed using the simulation-extrapolation method (SIMEX) [41]. For a subgroup of radiotherapy patients, ITT-data, as treated data and irradiation planning data are available. This subgroup will be used to estimate the variance and distribution of errors of the dose reconstruction data needed to perform SIMEX.

All analyses will be performed using SAS-Software 9.4 and R version 3.6.2.

## Results

The study covers data from 1244 cases and up to 4976 controls. 5596 patients were included once while 624 entered more than one time. A detailed list of FPNs, the mean age at diagnosis of FPN and SPN and the latencies between FPN and SPN can be found in Table 2. Data by SPN is described in Table 3. Of 1244 included SPN cases, 50.1% were female (*N* = 623), mean age at FPN diagnosis was 6.5 years (range 0–14 years), mean age at SPN diagnosis was 18.4 years with a range from 1 to 46 years. The mean latency was 11.9 years, while the maximum latency was 33 years. Most frequent FPNs of the cases were lymphoid leukemia (30.3%, *N* = 377, mean latency 11.7 years) and malignant neoplasms of the central nervous system (15,7%, *N* = 195, mean latency 11.8 years) (Table 2). The tumor type distribution of the FPN differs somewhat from the general childhood cancer distribution, because our inclusion criteria favor long-term survivors and primary diagnoses with relatively good survival. Most frequent single SPNs were AML (16.3%, *N* = 203, mean latency 4.4 years), tumors of the skin (including malignant melanoma, 12.3%, *N* = 153, mean latency 19.1 years), non-malignant (11,8%, *N* = 147, mean latency 17.4 years) and malignant brain tumors (10,8%, *N* = 134, mean latency 8.1 years), thyroid cancer (11.7%, *n* = 145, mean latency 14.2 years), and bone tumors (5.3%, *n* = 66, mean latency 9.5 years).

**Table 2:** Number of diagnoses, age at first primary neoplasm (FPN) for all sampled cases and controls (including backup controls), age at subsequent primary neoplasms (SPN) and latency between first and subsequent primary neoplasm in years for all sampled cases; by first neoplasm*

First neoplasm	*N*	Cases			Controls^a^		Total
		Age in years at FPN diagnosis	Age in years at SPN diagnosis	Latency (y) to SPN	*N*	Age in years at FPN diagnosis	
		Mean (Min–Max)	Mean (Min–Max)	Mean (Min–Max)		Mean (Min–Max)	
Lymphoid leukemia	377	5.6 (0–14)	17.4 (2–41)	11.7 (0.6–33.1)	1660	5.7 (0–14)	2037
Acute myeloid leukemia	55	6.9 (0–14)	16.5 (2–41)	9.8 (0.6–27.3)	216	6.6 (0–14)	271
Other leukemia	13	7.2 (1–14)	18.5 (3–32)	11.3 (0.7–28.4)	32	8.4 (0–14)	45
Hodgkin ‘s disease	127	11.3 (3–14)	27.7 (5–46)	16.3 (0.8–32.8)	395	11.0 (2–14)	522
Non-Hodgkin ‘s disease	100	7.8 (0–14)	19.7 (1–43)	11.8 (0.5–30.1)	369	8.5 (1–14)	469
Other lymphomas	0	**	**	**	10	7.2 (0–14)	10
Non-malignant CNS-tumors	33	7.4 (0–14)	18.2 (3–37)	10.8 (1.0–25.3)	335	7.4 (0–14)	368
Malignant CNS-tumors	195	5.7 (0–14)	17.5 (1–40)	11.8 (0.5–30.4)	437	6.7 (0–14)	632
Neuroblastomas	67	2.3 (0–13)	11.2 (1–35)	8.9 (1.1–30.6)	259	1.9 (0–14)	326
Retinoblastomas	33	0.7 (0–5)	12.5 (2–29)	11.7 (2.5–28.7)	138	1.4 (0–8)	171
Renal tumors	43	4.2 (0–13)	18.5 (4–40)	14.3 (0.7–32.3)	345	3.7 (0–14)	388
Liver tumors	4	**	**	7.4 (2.2–18.0)	27	2.4 (0–14)	31
Bone tumors	60	10.5 (5–14)	19.9 (8–39)	9.4 (0.6–24.7)	223	10.6 (0–14)	283
Soft tissue sarcomas	91	5.6 (0–14)	16.6 (2–41)	11.0 (1.2–31.9)	288	6.8 (0–14)	379
Germ cell tumors	35	8.3 (0–14)	21.2 (2–40)	13.0 (1.6–28.5)	190	7.2 (0–14)	225
Thyroid carcinomas	1	**	**	**	25	10.4 (5–14)	26
Skin tumors incl. Malignant Melanoma	1	**	**	**	6	9.0 (5–13)	7
Other tumors	9	8.7 (0–14)	18.3 (2–41)	9.6 (0.6–28.5)	21	9.0 (1–14)	30
Total	1244	6.5 (0–14)	18.4 (1–46)	11.9 (0.5–33.1)	4976	6.5 (0–14)	6220

*CNS* central nervous system, *SPN* subsequent primary neoplasm, *y* years, *N* number of cases

*Inclusion criteria for cases and controls: age at diagnosis of first neoplasm below 15, year of diagnosis of first neoplasm between 1980 and 2014, resident in Germany at the time of both the diagnosis of the first and second neoplasm, minimum latency between first and subsequent neoplasm: 0.5 years

**No results given as the number of patients was below 5

^a^Some patients were selected as case and as control for a different case (whose SPN occurred later in terms of age at diagnosis). Such individuals can account for multiple diagnosed tumors in this table, which have occurred at different periods in their lives

**Table 3:** Number of diagnoses, age at first primary neoplasm (FPN) for all sampled cases and controls (including backup controls), age at subsequent primary neoplasms (SPN) and latency between first and subsequent primary neoplasm in years for all sampled cases, by subsequent neoplasm*

Subsequent neoplasm	*N*	Cases			Controls ^a,b^		Total
		Age in years at FPN diagnosis	Age in years at SPN diagnosis	Latency (y) to SPN	*N*	Age in years at FPN diagnosis	
		Mean (Min–Max)	Mean (Min – Max)	Mean (Min–Max)		Mean (Min–Max)	
Lymphoid leukemia	47	6.2 (0–14)	11.6 (2–24)	5.5 (0.6–18.1)	188	6.2 (0–14)	235
Acute myeloid leukemia	203	6.4 (0–14)	10.8 (1–29)	4.4 (0.5–22.2)	812	6.4 (0–14)	1015
Other leukemia	6	10.8 (5–14)	14.8 (9–19)	3.9 (1.6–5.9)	24	10.9 (5–14)	30
Hodgkin ‘s disease	21	5.7 (0–14)	14.1 (4–31)	8.3 (1.6–20.2)	84	5.7 (0–14)	105
Non-Hodgkin ‘s disease	62	6.6 (0–14)	14.2 (2–39)	7.6 (0.7–29.1)	248	6.6 (0–14)	310
Other lymphomas	8	5.0 (0–11)	9.8 (3–19)	4.6 (0.7–11.3)	32	5.0 (0–11)	40
Non-malignant CNS-tumors	147	5.8 (0–14)	23.2 (5–42)	17.4 (0.5–30.4)	588	5.8 (0–14)	735
Malignant CNS-tumors	134	5.3 (0–14)	13.4 (2–32)	8.1 (1.6–28.1)	536	5.3 (0–14)	670
Neuroblastomas	8	2.0 (0–10)	6.4 (2–17)	4.4 (1.1–14.0)	32	2.0 (0–10)	40
Retinoblastomas	3	**	**	**	12	3.0 (1–6)	15
Renal tumors	14	4.3 (0–13)	14.1 (1–34)	9.7 (0.6–25.1)	56	4.3 (0–14)	70
Liver tumors	7	4.9 (2–9)	20.0 (4–37)	15.0 (1.7–32.3)	28	5.0 (2–9)	35
Bone tumors	66	4.3 (0–14)	13.8 (4–29)	9.5 (2.3–28.7)	264	4.4 (0–14)	330
Soft tissue sarcomas	62	5.1 (0–14)	16.0 (2–40)	11.0 (1.0–31.9)	248	5.1 (0–14)	310
Germ cell tumors	13	7.0 (0–14)	20.7 (10–38)	13.7 (2.8–24.6)	52	7.0 (0–14)	65
Thyroid carcinomas	145	6.9 (0–14)	21.1 (6–41)	14.2 (2.3–33.1)	580	6.9 (0–14)	725
Skin tumors incl. Malignant Melanoma	153	7.3 (0–14)	26.4 (5–43)	19.1 (1.5–32.8)	612	7.3 (0–14)	765
Other tumors	145	9.3 (0–14)	26.7 (4–46)	17.4 (0.6–32.1)	580	9.4 (0–14)	725
Total	1244	6.5 (0–14)	18.4 (1–46)	11.9 (0.5–33.1)	4976	6.5 (0–14)	6220

*CNS* central nervous system, *SPN* subsequent primary neoplasm, *y* years, *N* number of cases

*Inclusion criteria for cases and controls: age at diagnosis of first neoplasm below 15, year of diagnosis of first neoplasm between 1980 and 2014, resident in Germany at the time of both the diagnosis of the first and second neoplasm, minimum latency between first and subsequent neoplasm: 0.5 years **No results given as the number of patients was below 5

^a^Some patients were selected as case and as control for a different case (whose SPN occurred later in terms of age at diagnosis). Such individuals can account for multiple diagnosed tumors in this table, which have occurred at different periods in their lives

^b^Results refer to the subsequent neoplasm of the respective case

We had access to 124 treatment protocols included in the central protocol data base. 18 recent protocols were contributed to this data base by the STATT-SCAR study. 23 out of the 25 contacted clinical study centers provided treatment data or therapy arm information, although not always for all patients. Of the 49 hospitals, including all large pediatric oncology units in Germany, which were contacted, 28 provided at least part of the requested data. Overall, for 83% of all 1244 match groups, treatment data was available for the case and at least one control. Based on preliminary analyses more than 10% of the data were mixed source, about 75% ITT, and about 15% “as treated”-data, with some patients having data from multiple sources.

Based on preliminary analyses of the ITT-data, ca. 55% patients received both chemo- and radiotherapy, 42% received chemotherapy only, less than 1% received radiotherapy only, and ca. 2% received neither.

## Discussion

The STATT-SCAR-study is based on data of one of the largest and most complete (registration completeness > 95%) population-based childhood cancer registries worldwide [20]. Compared to a previous study [6], which analyzed 328 SPN cases and 639 controls, information from 12 additional years of follow-up and patient registration were added, which yielded 1244 SPN cases. This will be one of the largest studies of treatment related SPN risks. The number of cases is sufficient for additional subgroup analyses, e.g., for specific FPN or SPN entities and the separating of the effect of chemotherapy, radiotherapy and its combinations.

In contrast to similar studies focusing on 5-year survivors [42, 43], our cohort included all patients with a minimum latency of 6 months. Relevant early SPNs can be investigated, such as topoisomerase-inhibitor induced t-AMLs [44]. Another strength of our study was the availability of cumulative chemotherapy doses which allowed modeling continuous dose–response curves. Estimated organ doses of radiation therapy at the site of the SPN are available for future analyses, promising a more precise picture of the impact of irradiation, especially for more distant SPN.

The main limitation of our study is the large fraction of ITT-data (75%), for which individual protocol deviations remain unknown. We observed in the preliminary data, that individual dose reductions do occur, so ITT may overestimate the doses to an extent. For modeling the radiotherapy effect, exact information on tumor localization was often missing for both FPN and SPN. For patients with no precise information about the location of the tumor, irradiation areas have to be approximated. Surgery information was not considered at all. An additional limitation is the lack of systematic information on cancer predisposition syndromes. The information available is reliable, but incomplete. It is also biased and selective, as it was more likely reported with the SPN and not the primary, and more likely reported if the source of the SPN report was a pediatric oncology unit where SPNs at an early age were treated. We also need to acknowledge, that, even if this variable were systematically recorded at first diagnosis, this is necessarily retrospective data and the background information and diagnostic techniques on these syndromes increased considerably since 1980, rendering variability in the quality of reporting over the years.

We expect to gain a deeper insight into long-term effects of childhood cancer treatment. This can help to identify patient groups with an elevated risk for developing a SPN and therefore optimize screening and early detection for long-term survivors.

## Data Availability

Due to the German Data Protection law the data used in this study cannot be openly shared.
